# Association of a history of type 2 diabetes with short-term mortality in patients with decompensated cirrhosis

**DOI:** 10.3389/fendo.2026.1971737

**Published:** 2026-10-07

**Authors:** Honglei Ju, Guixiang Wang, Yujie Zhang, Bo Sun, Chiyi He, Jin Cheng, Wei Wang

**Affiliations:** 1 Department of Gastroenterology, Maanshan People’s Hospital, Maanshan, Anhui, China; 2 Department of Gastroenterology, The First Affiliated Hospital of Wannan Medical University (Yijishan Hospital of Wannan Medical University), Wuhu, Anhui, China; 3 Department of Gastroenterology, The Second Affiliated Hospital of Wannan Medical University, Wuhu, Anhui, China; 4 Department of Gastroenterology, The First People’s Hospital of Wuhu, Wuhu, Anhui, China; 5 Department of Gastroenterology, Affiliated Hospital of West Anhui Health Vocational College, Lu’an, Anhui, China

**Keywords:** decompensated liver cirrhosis, mortality, short-term outcome, stabilized inverse probability of treatment weights, type 2 diabetes mellitus

## Abstract

**Background:**

The association between type 2 diabetes mellitus (T2DM) and mortality in patients with liver cirrhosis remains controversial. This study aimed to evaluate whether a history of T2DM influences short-term all-cause mortality in hospitalized patients with decompensated liver cirrhosis (DLC).

**Methods:**

We conducted a retrospective study of hospitalized patients with DLC at three medical centers in China between November 2019 and June 2024. The study endpoint was the 6-month mortality. The association between T2DM and mortality was assessed using multivariable logistic regression, and stabilized inverse probability of treatment weighting (sIPTW) analysis was applied for sensitivity analysis.

**Results:**

Among 984 eligible patients, 239 (24.3%) had a history of T2DM. Patients with T2DM were older and had a higher prevalence of hypertension and coronary artery disease; however, no significant difference in 6-month mortality was observed between patients with and without T2DM. Multivariable logistic regression showed no independent association between a history of T2DM and mortality (OR = 0.80, 95% CI: 0.51–1.23), this result remained consistent using sIPTW analysis (OR = 0.78, 95% CI: 0.51–1.21).

**Conclusions:**

A history of T2DM may not be independently associated with short-term mortality in patients with DLC. These findings suggest that T2DM status may not be prioritized over liver-specific prognostic factors when assessing the short-term mortality risk in patients with DLC.

## 1 Introduction

Liver cirrhosis (LC) is an end-stage manifestation of chronic hepatic disorder. Globally, its escalating prevalence and mortality contribute to nearly 800,000 deaths annually, positioning it among the top ten global causes of mortality (1, 2). Patients progressing to decompensated cirrhosis (DLC) frequently develop life-threatening complications, including ascites, gastrointestinal hemorrhage, hepatic encephalopathy, and infections, which increase short-term mortality and impose significant healthcare and socioeconomic burdens (3).

The liver plays a pivotal role in glucose homeostasis by governing fundamental carbohydrate metabolic processes including glycogenolysis, gluconeogenesis, glycolysis, and glycogenesis (4). Epidemiological data indicate that 20–40% of patients with cirrhosis exhibit concomitant diabetes mellitus (5, 6). The association between diabetes and mortality risk in LC patients remains controversial. While several investigations have reported a significant increase in the mortality risk associated with diabetes (7, 8), other analyses fail to support such findings (9, 10). This unresolved discrepancy underscores the necessity for further research to examine the prognostic impact of diabetes mellitus in DLC populations. This study specifically investigated whether a history of type 2 diabetes mellitus (T2DM) was associated with short-term all-cause mortality in hospitalized patients with DLC.

## 2 Methods

### 2.1 Study cohort

This retrospective multicenter analysis evaluated the clinical records of DLC patients admitted to three Chinese tertiary hospitals (Maanshan People’s Hospital, Second Affiliated Hospital of Wannan Medical College, and Yijishan Hospital of Wannan Medical College) from November 2019 to June 2024. The three cohorts comprised 1,380 individuals hospitalized for a first-time DLC diagnosis. Diagnostic confirmation utilized either liver biopsy or an integrated assessment of characteristic clinical features, laboratory tests, radiological findings, and endoscopic observations (11). DLC was defined as decompensation onset in previously compensated cirrhotic patients, manifesting as ascites, variceal hemorrhage, hepatic encephalopathy, hepatorenal syndrome, or spontaneous bacterial peritonitis (SBP) (12). Exclusion criteria were as follows: (1) history of malignancy (n = 321), (2) type 1 diabetes mellitus (n = 2), (3) incomplete data (n = 47), and (4) loss to follow-up (n = 26). In total, 984 eligible patients were included in the analysis (**Figure 1**). The endpoint was 6-month all-cause mortality, which was determined via a medical record review or direct communication with patients or their relatives.

**Figure 1:**
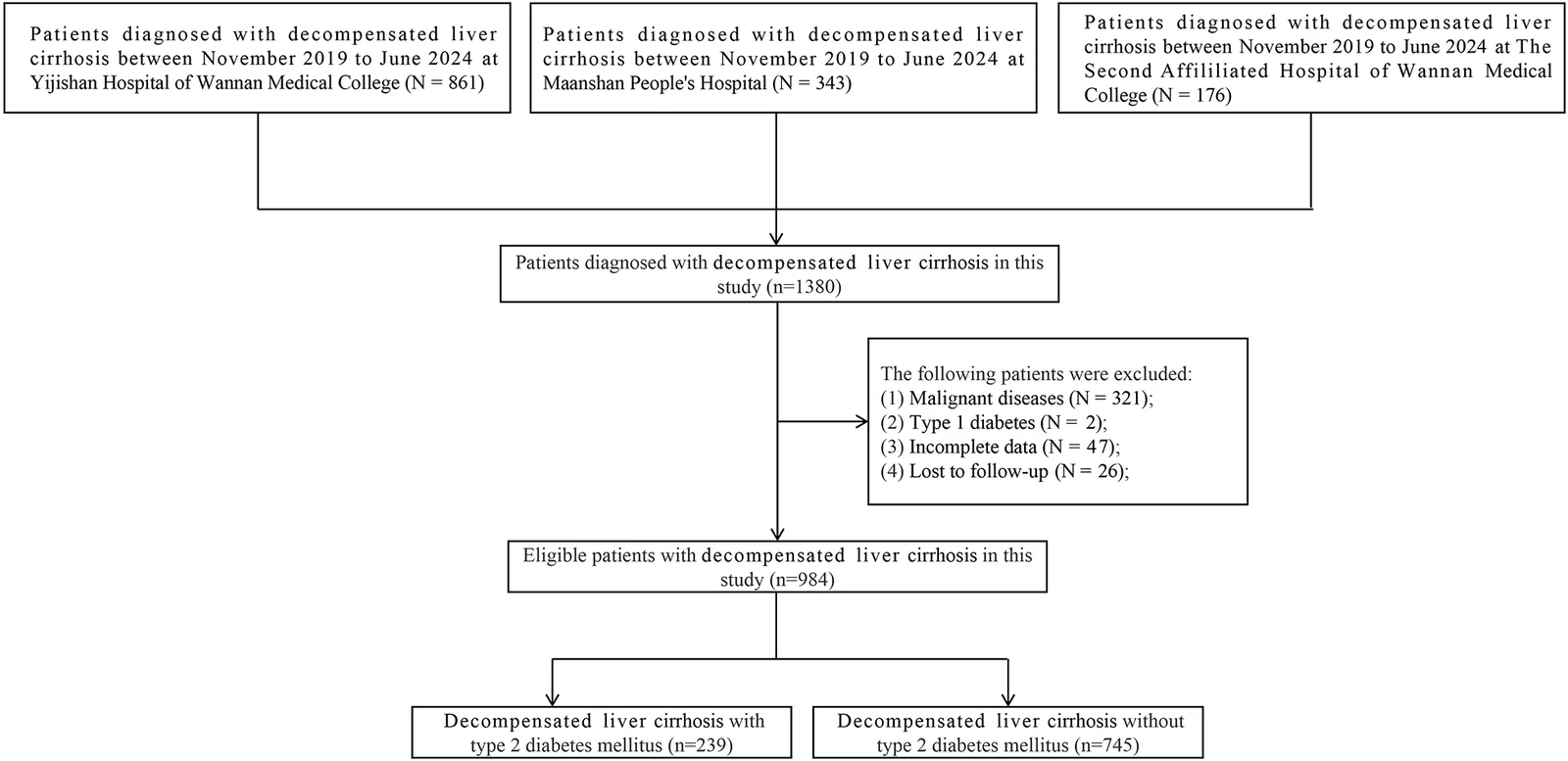
Flowchart of the patients selection process.

### 2.2 Clinical parameters

Patients’ electronic medical records provided the following variables: T2DM status, sex, age, hypertension, coronary artery disease (CAD), and chronic kidney disease (CKD). Cirrhosis etiology and decompensation manifestations (ascites, variceal bleeding, hepatorenal syndrome, hepatic encephalopathy, SBP) were documented. The model for End-Stage Liver Disease (MELD) score was calculated using an established methodology (13). Patients were classified as having a history of T2DM if either of the following criteria was met (14): (1) current use of antidiabetic medication or (2) a documented history of T2DM in the medical record. The estimated glomerular filtration rate (eGFR) was calculated according to previously published method (15). An eGFR < 60 mL/min/1.73 m² was used to define impaired baseline renal function (16).

### 2.3 Statistical analysis

Categorical data were summarized as counts (%) and analyzed using χ² tests. Continuous variables were reported as median (interquartile range [IQR]) using the Mann-Whitney U tests for group comparisons. The primary analysis employed multivariable logistic regression to assess the association between a history of T2DM and short-term mortality. In addition, a multivariable logistic model incorporating hypertension, CAD, CKD, age, and sex estimated the propensity scores for T2DM status (**Supplementary Table 1**). The stabilized inverse probability of treatment weights (sIPTW), using the above propensity scores, served as a sensitivity analysis for the primary analysis (17). To explore whether potential effects varied by etiological subtype, subgroup analyses were carried out. Interaction was formally tested using a likelihood ratio test, in which the primary model was compared with interaction models that incorporated product terms between T2DM status and each stratified variable. All analyses were performed using the R software (v.4.4.2), with a two-tailed significance threshold of p < 0.05.

## 3 Results

### 3.1 Patient characteristics

A total of 984 patients with DLC were included in this study, of whom 239 (24.3%) had a history of T2DM. The baseline characteristics of the patients are presented in **Table 1**. The median patient age was 65 years, and 57.6% of the patients were male. The prevalences of comorbidities were 30.8%, 5.3%, and 5.6% for hypertension, CAD, and CKD, respectively. Chronic hepatitis B virus infection was the predominant etiology (58.4%). The most common complications were ascites (72.3%), followed by variceal bleeding (38.3%), SBP (9.2%), hepatic encephalopathy (8.0%), and hepatorenal syndrome (4.4%). Six-month all-cause mortality was 15.1%, with a baseline median MELD score of 11.9. T2DM patients exhibited significantly higher age and had greater hypertension and CAD prevalence than non-T2DM patients. However, no significant difference in short-term mortality was observed between the two groups. Among T2DM patients, disease management strategies included dietary alone (12.6%, n=30), oral hypoglycemic agents (40.2%, n=96), and insulin therapy (53.1%, n=127).

**Table 1:** Clinical characteristics of decompensated liver cirrhosis patients by T2DM.

Variables	Total	No history of T2DM	History of T2DM	P value
	(n = 984)	(n = 745)	(n = 239)	
Sex (n, %)				0.914
male	567 (57.6%)	430 (57.7%)	137 (57.3%)	
female	417 (42.4%)	315 (42.3%)	102 (42.7%)	
Age (years)	65.0 (54.0-72.8)	63.0 (53.0-72.0)	66.0 (56.0-73.0)	0.015
Hypertension (n, %)	303 (30.8%)	180 (24.2%)	123 (51.5%)	<0.001
Coronary artery disease (n, %)	52 (5.3%)	29 (3.9%)	23 (9.6%)	0.001
Chronic kidney disease (n, %)	55 (5.6%)	38 (5.1%)	17 (7.1%)	0.239
Modes of decompensation (n, %)				
Ascites	711 (72.3%)	543 (72.9%)	168 (70.3%)	0.436
Variceal bleeding	377 (38.3%)	283 (38.0%)	94 (39.3%)	0.710
HE	79 (8.0%)	49 (6.6%)	30 (12.6%)	0.003
HRS	43 (4.4%)	31 (4.2%)	12 (5.0%)	0.572
SBP	91 (9.2%)	68 (9.1%)	23 (9.6%)	0.818
MELD score	11.9 (9.2-15.5)	11.8 (9.1-15.3)	12.0 (9.5-16.3)	0.240
Mortality (n, %)	149 (15.1%)	114 (15.3%)	35 (14.6%)	0.805
Etiology (n, %)				
HBV	575 (58.4%)	425 (57.0%)	150 (62.8%)	0.119
HCV	60 (6.1%)	48 (6.4%)	12 (5.0%)	0.424
Alcoholism	71 (7.2%)	55 (7.4%)	16 (6.7%)	0.721
Others	278 (28.3%)	217 (29.1%)	61 (25.5%)	0.282
Antidiabetic treatment^*^				
Dietary control only	/	/	30 (12.6%)	
Insulin therapy	/	/	127 (53.1%)	
Oral hypoglycemic agents	/	/	96 (40.2%)	

Data are expressed as number, median (25th–75th percentiles), or frequency [percentage (%)]. *Notes:* T2DM, type 2 diabetes mellitus; HE, hepatic encephalopathy; HRS, hepatorenal syndrome; SBP, spontaneous bacterial peritonitis; MELD, model for endstage liver disease; HBV, hepatitis B virus; HCV, hepatitis C virus. ^*^Treatment categories were not mutually exclusive; some patients received multiple therapies.

### 3.2 Association between T2DM and short-term mortality in patients with DLC

Multivariable logistic regression demonstrated no independent association between a history of T2DM and mortality risk (adjusted odds ratio [OR] = 0.80, 95% confidence interval [CI]: 0.51–1.23). After adjusting for covariates, a consistent result was observed in the propensity score analysis using sIPTW (adjusted OR = 0.78, 95% CI: 0.51–1.21) (**Table 2**).

**Table 2:** Association between a history of T2DM and short-term mortality in patients with DLC across crude, multivariable, and propensity score analyses.

Analysis (History of T2DM(yes/no))	Odds ratio	95% CI
Crude analysis	0.95	0.62-1.42
Multivariable analysis^*^	0.80	0.51-1.23
Analysis with stabilized inverse probability weighting^#^	0.78	0.51-1.21

* Multivariable logistic regression–derived odds ratios adjusted for age, sex, hypertension, coronary artery disease, and chronic kidney disease.

# Odds ratios derived from the multivariate logistic regression model were adjusted for age, sex, hypertension, coronary artery disease, and chronic kidney disease, with a stabilized inverse probability of treatment weighting based on propensity scores.

T2DM, type 2 diabetes mellitus; CI, confidence interval.

To further assess the consistency of the association across cirrhosis etiologies, we performed etiology-stratified subgroup analyses. The association between T2DM status and mortality risk was not significant and did not significantly differ across etiological subgroups (P for interaction = 0.584) (**Supplementary Table 2**). In addition, we evaluated differences in mortality and complications according to baseline renal function. Patients with impaired baseline renal function had a higher mortality than those without impaired baseline renal function (30.0% vs. 9.5%, P < 0.001) and higher frequencies of hepatic encephalopathy and hepatorenal syndrome (**Supplementary Table 3**).

Further, we performed several sensitivity analyses. First, we explored the association between a history of T2DM and outcome in patients with severely decompensated cirrhosis (MELD score >20). The results showed that a history of T2DM was not statistically significantly associated with six-month all-cause mortality after multivariable adjustment (adjusted OR, 0.63; 95% CI, 0.21–1.76). Second, based on the primary analysis, we further adjusted for HE or MELD score, respectively. The results showed that a history of T2DM was not statistically significantly associated with six-month all-cause mortality [adjusted OR (additionally adjusted for HE), 0.69; 95% CI, 0.43–1.08; adjusted OR (additionally adjusted for MELD score), 0.75; 95% CI, 0.47–1.16].

## 4 Discussion

Our findings demonstrate that a history of T2DM was not statistically significantly associated with short-term mortality risk in patients with DLC.

Previous studies have reported inconsistent findings regarding the association between T2DM and mortality in patients with cirrhosis. Some studies have suggested that T2DM may increase the mortality risk. A Korean cohort study identified T2DM as an independent determinant of mortality in alcohol-related cirrhosis with acute decompensation (7). A database analysis from Taiwan documented T2DM significantly association with increased all-cause mortality risk in patients with compensated cirrhosis (8). A Mexican prospective cohort (110 compensated cirrhosis patients) revealed that a history of diabetes substantially increased mortality risk (18). A Germany study identified diabetes as an independent predictor of poorer survival (19). A study conducted among Singaporean Chinese individuals indicated that diabetes is an independent risk factor for mortality (20). Conversely, the findings of multiple studies are inconsistent with the aforementioned conclusions. Wlazlo et al. observed that diabetes was not independently associated with mortality in patients with cirrhosis (6). Australian hospitalized LC cohort analysis found that T2DM was not independently associated with mortality (21). Guevara et al. reported that diabetes was not independently associated with an increased risk of mortality in patients with cirrhosis and ascites (9). A recent Indian study showed that hepatogenous diabetes was not independently associated with 90-day mortality in outpatients with DLC (22). The inconsistencies observed in previous studies may be related to differences in study populations (compensated or decompensated status), variations in etiology, and divergent outcome definitions (short-term or long-term mortality).

This study had several limitations. First, although the sample size was substantial, the retrospective design necessitates prospective validation. Second, the temporal sequence of these diseases (LC and T2DM) remains undetermined. Third, residual confounding could not be fully ruled out despite multivariable adjustment. Fourth, data on the duration of diabetes were unavailable. Fifth, detailed cardiac function assessments were unavailable, precluding evaluation of the relationship between impaired systolic or diastolic function and T2DM in patients with DLC; comprehensive inclusion of cardiac function-related data in future studies would be beneficial for further investigation of this issue. Sixth, detailed data on bacterial and fungal infections during follow-up were not systematically available, precluding a reliable comparison of outcome based on the presence or absence of bacterial or fungal infections during follow-up between the T2DM and non-T2DM groups in patients with DLC.

## 5 Conclusions

A history of T2DM may not be independently associated with an increased risk of short-term all-cause mortality in patients with DLC. Future investigations should investigate the association between a history of T2DM and long-term outcomes to establish clinically applicable risk-stratification models.

## Data Availability

The raw data supporting the conclusions of this article will be made available by the authors, without undue reservation.
