# Supplementary material for: Association of a history of type 2 diabetes with short-term mortality in patients with decompensated cirrhosis
Source: Front Endocrinol (Lausanne). 2026 Oct 7;17:1971737. doi: 10.3389/fendo.2026.1971737 (PMC13683143; doi:10.3389/fendo.2026.1971737)
Supplement: Supplementary file 1 [file Table1.docx]

Supplementary Table 1 Baseline clinical characteristics of patients with decompensated liver cirrhosis according to T2DM history before and after stabilized inverse probability of treatment weighting.

| Variables | Before sIPTW | | | | After sIPTW | | | |
| --- | --- | --- | --- | --- | --- | --- | --- | --- |
|  | No history of T2DM | History of T2DM | SMD | *p* | No history of T2DM | History of T2DM | SMD | *p* |
|  | (n = 745) | (n =239) |  |  | (n = 745.05) | (n = 238.88) |  |  |
| Sex (n, %) |  |  | 0.008 | 0.914 |  |  | 0.005 | 0.950 |
| Male | 430 (57.7%) | 137 (57.3%) |  |  | 428.9 (57.6) | 13.6.9 (57.3) |  |  |
| Female | 315 (42.3%) | 102 (42.7%) |  |  | 316.2 (42.4) | 102.0 (42.7) |  |  |
| Age (years) | 63.0 (53.0-72.0) | 66.0 (56.0-73.0) | 0.200 | 0.015 | 63.1±13.2 | 63.2±11.4 | 0.008 | 0.913 |
| Hypertension (n, %) |  |  | 0.587 | <0.001 |  |  | <0.001 | 0.999 |
| Yes | 180 (24.2%) | 123 (51.5%) |  |  | 229.4 (30.8) | 73.6 (30.8) |  |  |
| No | 565 (75.8%) | 116 (48.5%) |  |  | 515.6 (69.2) | 165.3 (69.2) |  |  |
| Coronary artery disease (n, %) |  |  | 0.230 | 0.001 |  |  | 0.002 | 0.973 |
| Yes | 29 (3.9%) | 23 (9.6%) |  |  | 39.5 (5.3) | 12.8 (5.4) |  |  |
| No | 716 (96.1%) | 216 (90.4%) |  |  | 705.6 (94.7) | 226.1 (94.6) |  |  |
| Chronic kidney disease (n, %) |  |  | 0.084 | 0.239 |  |  | 0.011 | 0.883 |
| Yes | 38 (5.1%) | 17 (7.1%) |  |  | 41.9 (5.6) | 14.1 (5.9) |  |  |
| NO | 707 (94.9%) | 222 (92.9%) |  |  | 703.1 (94.4) | 224.8 (94.1) |  |  |

*Note:* sIPTW, stabilized inverse probability of treatment weights; T2DM, type 2 diabetes mellitus; SMD, Standardized Mean Difference.
