# Supplementary material for: Association of a history of type 2 diabetes with short-term mortality in patients with decompensated cirrhosis
Source: Front Endocrinol (Lausanne). 2026 Oct 7;17:1971737. doi: 10.3389/fendo.2026.1971737 (PMC13683143; doi:10.3389/fendo.2026.1971737)
Supplement: Supplementary file 2 [file Table2.docx]

Supplementary Table 2. Subgroup analyses were conducted to explore potential effects within specific etiological subtypes on six-month all-cause mortality in decompensated liver cirrhosis patients with or without a history of T2DM.

| Subgroup | No./Events | OR (95% CI) | P for interaction |
| --- | --- | --- | --- |
| Etiology |  |  | 0.584 |
| HBV | 575/92 | 0.63 (0.34-1.12) |  |
| HCV | 60/5 | 0.08 (0.03-1.82) |  |
| Alcoholism | 71/16 | 1.37 (0.31-5.47) |  |
| Others | 278/36 | 1.21 (0.50-2.72) |  |

Odds ratios derived from the multivariate logistic regression model were adjusted for age, sex, hypertension, coronary artery disease, and chronic kidney disease

*Notes:* OR, odds ratio; CI, confidence interval; HBV, hepatitis B virus; HCV, hepatitis C virus.
