# Supplementary material for: Association of a history of type 2 diabetes with short-term mortality in patients with decompensated cirrhosis
Source: Front Endocrinol (Lausanne). 2026 Oct 7;17:1971737. doi: 10.3389/fendo.2026.1971737 (PMC13683143; doi:10.3389/fendo.2026.1971737)
Supplement: Supplementary file 3 [file Table3.docx]

Supplementary Table 3. Mortality and complications in patients with a history of T2DM stratified by baseline eGFR.

| Variables | T2DM with eGFR < 60 mL/min/1.73 m² (n=60) | T2DM with eGFR >= 60 mL/min/1.73 m² (n=179) | p value |
| --- | --- | --- | --- |
| Mortality (n, %) | 18 (30.0%) | 17 (9.5%) | < 0.001 |
| Ascites (n, %) | 42 (70.0%) | 126 (70.4%) | 0.954 |
| Varicealbleeding (n, %) | 20 (33.3%) | 74 (41.3%) | 0.272 |
| HE (n, %) | 13 (21.7%) | 17 (9.5%) | 0.014 |
| HRS (n, %) | 12 (20.0%) | 0 (0%) | < 0.001 |

*Notes:* Data are presented as number (percentage). eGFR, estimated glomerular filtration rate; T2DM, type 2 diabetes mellitus; HE, hepatic encephalopathy; HRS, hepatorenal syndrome; SBP, spontaneous bacterial peritonitis.

.
